# Association of cerebral small vessel disease burden with brain structure and cognitive and vascular risk trajectories in mid-to-late life

**DOI:** 10.1177/0271678X211048411

**Published:** 2021-10-05

**Authors:** Michelle G Jansen, Ludovica Griffanti, Clare E Mackay, Melis Anatürk, Luca Melazzini, Ann-Marie G de Lange, Nicola Filippini, Enikő Zsoldos, Kim Wiegertjes, Frank-Erik de Leeuw, Archana Singh-Manoux, Mika Kivimäki, Klaus P Ebmeier, Sana Suri

**Affiliations:** 1 Donders Centre for Cognition, Donders Institute for Brain, Cognition and Behaviour, Radboud University, Nijmegen, the Netherlands; 2 Department of Neurology, Donders Institute for Brain, Cognition and Behavior, Radboud University Medical Center, Nijmegen, the Netherlands; 3 Department of Psychiatry, 6396 University of Oxford, University of Oxford, Oxford, UK; 4 Wellcome Centre for Integrative Neuroimaging (Oxford Centres for Functional MRI of the Brain & Human Brain Activity) University of Oxford, Oxford, UK; 5 Centre for Medical Image Computing, Department of Computer Science, 4919 University College London, University College London, London, UK; 6 Department of Biomedical Sciences for Health, Università degli Studi di Milano, Milan, Italy; 7 Department of Psychology, 6305 University of Oslo, University of Oslo, Oslo, Norway; 8 IRCCS San Camillo Hospital, Venice, Italy; 9 Department of Epidemiology and Public Health, 4919 University College London, University College London, London, UK; 10 INSERM, Epidemiology of Ageing and Neurogenerative Diseases, Université de Paris, Paris, France

**Keywords:** Ageing, cardiovascular risk, cognition, magnetic resonance imaging, small vessel disease

## Abstract

We characterize the associations of total cerebral small vessel disease (SVD) burden with brain structure, trajectories of vascular risk factors, and cognitive functions in mid-to-late life. Participants were 623 community-dwelling adults from the Whitehall II Imaging Sub-study with multi-modal MRI (mean age 69.96, SD = 5.18, 79% men). We used linear mixed-effects models to investigate associations of SVD burden with up to 25-year retrospective trajectories of vascular risk and cognitive performance. General linear modelling was used to investigate concurrent associations with grey matter (GM) density and white matter (WM) microstructure, and whether these associations were modified by cognitive status (Montreal Cognitive Asessment [MoCA] scores of < 26 vs. ≥ 26). Severe SVD burden in older age was associated with higher mean arterial pressure throughout midlife (β = 3.36, 95% CI [0.42-6.30]), and faster cognitive decline in letter fluency (β = −0.07, 95% CI [−0.13–−0.01]), and verbal reasoning (β = −0.05, 95% CI [−0.11–−0.001]). Moreover, SVD burden was related to lower GM volumes in 9.7% of total GM, and widespread WM microstructural decline (FWE-corrected *p* < 0.05). The latter association was most pronounced in individuals who demonstrated cognitive impairments on MoCA (MoCA < 26; *F_3,608_* = 2.14, *p* = 0.007). These findings highlight the importance of managing midlife vascular health to preserve brain structure and cognitive function in old age.

## Introduction

Cerebral small vessel disease (SVD) refers to a series of pathological processes damaging the small perforating arterioles of the brain.^1^ Conventional MRI markers of SVD include, amongst others, white matter hyperintensities (WMH), enlarged perivascular spaces (EPVS), cerebral microbleeds (CMB), and lacunes.^1,2^ The total SVD burden score combines the visual ratings of these markers, providing a more comprehensive measure of SVD than each individual component alone.^3,4^

Accumulating evidence suggests that controlling vascular risk factors (e.g., blood pressure, obesity) earlier in the lifespan may preserve structural brain health in older ages and hence contribute to preventing dementia.^5–9^ However, previous research investigating longitudinal correlates of the SVD burden score have been limited to stroke patients or solely examined sociodemographic factors (e.g., intelligence at age 11).^10,11^ SVD burden varies considerably between healthy older adults^10^ and contributes to several other brain structural impairments.^1^ However, the relationship between total SVD scores and brain morphology is less clear.^12^ Studies investigating the longitudinal vascular and cognitive correlates of SVD, together with structural brain associations, may provide further insights into the stage in life when interventions designed to promote healthy aging could ideally be administered.

We investigated the associations of total SVD burden with (1) up to 25-year retrospective trajectories of vascular risk factors (mean arterial pressure [MAP]; body mass index [BMI]; Framingham Stroke Risk Score [FSRS]) and cognitive decline on several domains and (2) concurrent grey matter (GM) density and white matter (WM) microstructure in 623 elderly individuals from the Whitehall II Imaging cohort. We also examined whether the associations of SVD burden with brain structure were moderated by cognitive status in older age.

## Methods

### Study design and participants

Participants were selected from the Whitehall II Imaging cohort, a sub-study of the prospective Whitehall II cohort, established in University College London in 1985. Whitehall II participants have received detailed clinical follow-ups for up to 30 years in 1991–1994 (Wave 3), 1997–1999 (Wave 5), 2002–2004 (Wave 7), 2007–2009 (Wave 9), 2012–2013 (Wave 11), and 2015–2016 (Wave 12). For the Imaging Sub-study, 800 participants (60–85 years old) were randomly selected from the Whitehall II Wave 11 cohort and underwent a detailed neuropsychological assessment and multi-modal 3 T brain MRI scans at the University of Oxford between 2012–2016 (MRI Wave).^13^ A total of 623 participants were included after excluding participants with missing data for variables of interest in three or more waves (N = 36), the occurrence of gross MRI abnormalities (e.g., large strokes, cysts, tumours, hydrocephalus which failed the MRI pre-processing pipelines; N = 28), and missing or insufficient quality of MRI images for analysis (e.g., motion artefacts; N = 88). A detailed description and flowchart of participant inclusion is presented in Supplementary Methods and Figure S1. The Whitehall II cohort profile and the Imaging Sub-study protocol have been described previously.^13,14^

### Standard protocol approvals, registrations, and patient consents

Participants gave informed written consent in a procedure approved by the University of Oxford Central University Research Ethics Committee (Application reference: MS IDREC-C1- 2011–71) for participating at the Whitehall II MRI Substudy and the University College London Medical School Committee on the Ethics of Human Research (reference: 85/0938) at each wave of the full Whitehall II cohort study, both in accordance with the Helsinki Declaration of 1975 (and as revised in 1983).

### Demographics

Demographic variables included age, sex, ethnicity, years of full-time education (self-reported at MRI Wave), and highest employment grade at Wave 3 in 1991–1994 to indicate socio-economic status (high =managers/administrators, intermediate = professionals/executives, low = clerical/support staff).

### Neuroimaging

MRI scans were acquired on a 3 T Siemens Magnetom Verio scanner (Erlangen, Germany) between April 2012–December 2014 (N = 427). After a scanner upgrade, the remaining 196 scans were acquired on a 3 T Siemens Magnetom Prisma scanner (Erlangen, Germany) between June 2015–April 2016. We examined the following sequences: high-resolution T1-weighted images; fluid-attenuated recovery imaging (FLAIR); T2*-weighted images; and diffusion weighted imaging. Sequences were closely matched between the two scanners and acquisition parameters are described in Table S1, and elsewhere.^6,13^

### Total SVD burden

Visual ratings were performed by experienced raters in accordance with the Standards for Reporting Vascular Changes on Neuroimaging (STRIVE) criteria, and were blind to the clinical, cognitive, and other derived MRI variables.^2^ Periventricular and deep WMH were rated on FLAIR images, lacunes on T1 and FLAIR images, EPVS on T1 images, and CMB on T2* images. Details on visual ratings and intra-rater reliability are provided in Supplementary Methods. These ratings were used to calculate total SVD scores to express SVD severity on an ordinal scale from 0–4; 1 point each was provided for a Fazekas score of deep WMH ≥2 and/or periventricular WMH > 2, CMB count ≥1, lacunes ≥1, and EPVS ≥11 in the basal ganglia of one hemisphere.^3,4^ As only 2% of the sample (N = 13) had a SVD burden score of 4, we merged groups with scores of 3 and 4.

### Vascular risk factors

Vascular risk factors were assessed six times at 5-year intervals from 1991–1994 (Wave 3) to 2015–2016 (Wave 12), using questionnaires and clinical examinations (for details, see Supplementary Methods). Based on the literature linking vascular risk factors and cognitive decline,^6–8^ we selected three measures of vascular risk for the longitudinal trajectory analysis: MAP [(systolic BP + 2 × diastolic BP)/3], BMI (kg/m^2^) and the FSRS. The FSRS predicts the likelihood of a stroke within 10 years, calculated with an algorithm which combines age, sex, systolic blood pressure, self-reported use of antihypertensives, diabetes, current smoking, current or previous atrial fibrillation, left ventricular hypertrophy, and cardiovascular disease.^15^

At the MRI Wave, weekly alcohol consumption was self-reported, and metabolic equivalents of task (METs) per week for moderate-to-vigorous activity were calculated using the self-administered Community Healthy Activities Model Program for Seniors (CHAMPS) questionnaire.^16^

### Cognitive function

Cognitive function was evaluated five times at 5-year intervals from 1997–1999 (Wave 5) to 2015–2016 (Wave 12), including letter fluency (number of ‘S’ words listed in one minute), semantic fluency (category ‘animals’), short-term memory (recall of a list of 20 words in two minutes), and verbal and numerical reasoning as assessed with the Alice Heim 4-I test.^17^

We also used the MoCA, evaluated only at the MRI Wave, to assess cognitive status outcome. We classified cognitive impairments as performance below the traditional established screening cut-offs (MoCA scores < 26).^18^ Detailed information on the above measures is provided in Supplementary Methods.

#### MRI outcomes

MRI scans were analysed using FMRIB Software Library v6.0 (FSL; https://fsl.fmrib.ox.ac.uk/). Detailed information on pre-processing pipelines and harmonization of images acquired from the Verio and Prisma scanner have been described in previous work,^6,13,19^ and in Supplementary Methods. Briefly, pre-processed T1 images were analysed using voxel-based morphometry (FSL-VBM) to produce GM density maps for each participant. Pre-processing of DTI images produced four maps for each participant: fractional anisotropy (FA), mean diffusivity (MD), radial diffusivity (RD), and axial diffusivity (AD), which are widely used to estimate axonal and myelin integrity.^20^ We used Tract-Based Spatial Statistics (TBSS) to create skeletonized (thinned) FA, MD, RD and AD maps. All maps were concatenated in 4D files for subsequent voxel-wise statistics. Measures of mean global FA, MD, RD and AD were extracted from the respective skeletonized maps. WMH were segmented on FLAIR images using the supervised FMRIB’s Brain Intensity AbNormality Classification Algorithm (BIANCA) tool.^21^ Binarized WMH maps were generated by selecting voxels that exceeded a probability of 0.9 of being WMH on the BIANCA output, and used to quantify WMH volumes as % of total brain volume.

### Statistical analysis

All statistical analyses were performed using R version 3.6.1. Effects were considered significant when *p* < 0.05 (two-tailed). We tested continuous variables for normality using the Shapiro-Wilks test. Participant characteristics and physiological measurements were compared between SVD groups (0–3) using one-way analysis of variance (ANOVA), Kruskall-Wallis test, and Chi-square test as appropriate. Effect sizes were calculated where possible and expressed with Cohen’s f^2^ (f^2^ = 0.02 small; f^2^ = 0.15 medium; f^2^ = 0.35 large).

Linear mixed effect models (LME) with random slopes and intercepts were used to investigate whether individuals in the four SVD burden groups differed in baseline measures and longitudinal trajectories of vascular risk factors (MAP, BMI, FSRS) and cognition (letter and semantic fluency, memory, verbal and numerical reasoning). A complete description of the LME models is provided in Supplementary Methods. We tested for non-normality of the residuals by using the Shapiro-Wilks test. FSRS was log-transformed due to the skewed distribution of the standardized residuals. A binary covariate was added to control for the effects of MRI scanner model. Further, we corrected for the effects of age, sex, and education, by scaling these variables and incorporating their main effects and interactions with the time terms. A linear model best described MAP and BMI trajectories; however, including an orthogonalized polynomial quadratic time term improved model fit for FSRS and cognitive trajectories (all *p* < 0.05).^22,23^ This quadratic time term expresses non-linear (exponential) changes in the LME model without inducing collinearity.^24^ We performed Bonferroni corrections for multiple comparisons across the 5 cognitive measures. Accordingly, results surpassing a strict significance threshold (*p* < 0.01) are discussed in detail, whereas those 0.01 < *p* < 0.05 are interpreted with caution.

Voxel-wise associations of SVD burden with GM density and DTI-derived metrics (FA, MD, RD, AD) were investigated with general linear modelling (GLM) and permutation-based non-parametric testing (5000 permutations) using the FSL Randomize Tool. A separate voxel-wise GLM was performed for each DTI metric, and included the following covariates: age, sex, education, MRI scanner model, antihypertensive use, BMI, and MAP measured at the time of MRI. Analyses were corrected for multiple voxel-wise comparisons using family-wise error (FWE) corrections and reporting threshold-free cluster enhancement (TFCE) statistics. We applied a Bonferroni correction for multiple comparisons across the 4 DTI metrics, accepting a strict significance threshold of *p* < 0.0125. To examine whether the effects of SVD burden on DTI metrics were driven by the presence of WMH, we performed a sensitivity analysis which included the WMH spatial maps as voxel-wise confounds in the model.

We also examined whether the associations of SVD burden with global measures of GM and WM microstructure were moderated by cognitive status (MoCA < 26 vs. MoCA ≥ 26) at the time of MRI. Multivariate analysis of covariance (MANCOVA) was performed with cognitive status and SVD burden as independent variables, and global GM, mean global FA, MD, RD, and AD as dependent variables, and the same covariates as above.

### Data availability

The study follows Medical Research Council data sharing policies (https://mrc.ukri.org/research/policies-and-guidance-for-researchers/data-sharing/), and data from the Whitehall II Study and Whitehall II Imaging Sub-study are accessible via application to the Dementias Platform UK (https://portal.dementiasplatform.uk/).

## Results

At the time of MRI, the mean age of the 623 participants was 69.96 years (SD = 5.18), 494 (79%) were men, and 594 (95%) Caucasian, reflecting the demographics of the parent Whitehall II study. Sample demographics, physiological measurements, and the distribution of the total SVD score at the time of MRI (2012–2016) are presented in Table 1.

**Table 1.** Participant characteristics and associations at the MRI wave (2012–2016).

		Total SVD score				
	All (N = 623)	0 (N = 209)	1 (N = 206)	2 (N = 145)	3 (N = 63)	*p*
SVD markers						
Lacunes, N (%)	86 (14%)	0 (0%)	17 (8%)	32 (22%)	37 (59%)	
EPVS, N (%)	203 (33%)	0 (0%)	57 (28%)	90 (62%)	56 (89%)	
WMH, N (%)	209 (34%)	0 (0%)	53 (26%)	97 (67%)	59 (94%)	
CMB, N (%)	200 (32%)	0 (0%)	79 (38%)	71 (49%)	50 (79%)	
Participant characteristics						
Age, median (IQR)	69 (66.01–73.28)	67.39 (65.5–70.58)	68.12 (65.85–72.64)	71.2 (67.84–75.1)	72.18 (69–77.86)	**<0.001**
Age, mean (SD)	69.96 (5.18)	68.45 (4.46)	69.37 (4.93)	71.58 (5.23)	73.22 (5.76)	**<0.001**
Female, N (%)	129 (21%)	41 (20%)	31 (15%)	39 (27%)	18 (29%)	**0.02**
Caucasian ethnicity, N (%)	587 (94%)	198 (95%)	193 (94%)	134 (92%)	62 (98%)	0.38
Years of education, median (IQR)	13 (12–17)	14 (12–17)	13.5 (12–17)	13 (12–17)	13 (11.25–16)	0.19
Highest employment grade, N (%)	319 (51%)	105 (50%)	103 (50%)	83 (58%)	28 (44%)	0.36
Intermediate employment grade, N (%)	264 (42%)	90 (43%)	91 (63%)	54 (37%)	29 (46%)	0.49
Lowest employment grade, N (%)	29 (4%)	9 (4%)	10 (5%)	6 (4%)	4 (6%)	0.89
MoCA, median (IQR)	28 (26–29)	28 (26–29)	27 (26–29)	28 (26–29)	28 (26.5–29)	0.41
BMI, median (IQR)	25.34 (23.34–28.07)	25.39 (23.13–28.29)	25.38 (23.19–27.9)	25.54 (23.46–28.22)	24.94 (23.46–27.87)	0.95
MAP, mean (sd)	98.84 (11.82)	97.67 (12.31)	99.38 (11.03)	98.09 (12.19)	102.39 (11.62)	**0.02**
Diabetes, N (%)	58 (9%)	16 (8%)	18 (9%)	20 (14%)	4 (6%)	0.18
Current smoking, N (%)	20 (3%)	7 (3%)	7 (3%)	5 (3%)	1 (2%)	0.90
Alcohol use, median (IQR)	11.22 (4.09–20.36)	10.5 (4.25–19)	12.02 (4.25–23.23)	9.5 (2.10–16.82)	12.60 (3.87–20.61)	0.09
Antihypertensive use, N (%)	192 (31%)	47 (22%)	62 (30%)	54 (37%)	29 (46%)	**<0.001**
Physical activity, MET, median, (IQR)	1155 (495–2062.50)	1222.5 (525–2082.5)	1290 (592.5–2302.5)	982.5 (395.5–1792.5)	840 (300–1845)	**0.02**

SVD: cerebral small vessel disease; EPVS: enlarged perivascular spaces; WMH: white matter hyperintensities; CMB: cerebral microbleeds; BMI: body mass index; BP: blood pressure, MET: metabolic equivalent task. Information on employment grade was missing in 2% (N = 11), alcohol use in 3% (N = 21), physical activity in 0.003% (N = 2).

Individuals with higher SVD burden scores were older, more often female, demonstrated higher MAP, were more likely to be on antihypertensive treatment, and engaged in less physical activity. Participants’ demographics and vascular risk factors at the five follow-up waves are shown in Table S2. The average follow-up time from Wave 3 to Wave 12 was 23.5 years (SD = 0.57, interquartile range [IQR] 23.17–23.91). The average time from Wave 3 to the MRI Wave was 22.15 years (SD = 1.40, IQR 21.02–23.39), and from Wave 5 to MRI was 16.38 years (SD = 1.30, IQR 15.27–17.56). The demographic characteristics of the 623 included participants did not differ from the starting sample of N = 775 with scans from the Whitehall II Imaging Sub-Study (Table S3). Thirty-four percent of the sample demonstrated total SVD scores of 0, followed by scores of 1 (33%), 2 (23%) and 3 (10%). Lacunes were the least frequently observed SVD feature (14%), whereas EPVS, WMH and CMB were all equally prevalent (32–34%; see Figure S2 for a Venn-diagram).

**Figure 1.**
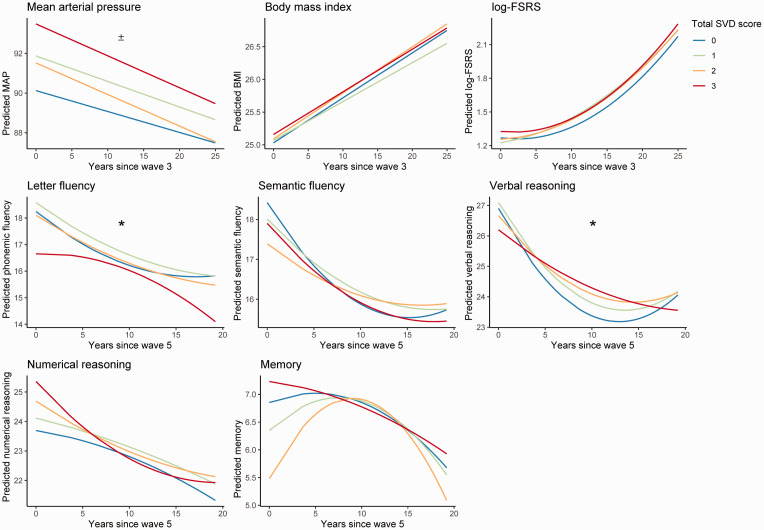
Predicted longitudinal trajectories for vascular risk and cognitive performance. Each figure depicts the predicted trajectories of the dependent variable of interest based on the linear mixed effect models. Measures for mean arterial pressure (MAP) and log-Framingham stroke risk score (log-FSRS) were obtained between 1995 and 2016. Measures on cognitive performance were obtained between 1997 and 2016. ± indicates significant main effects of SVD burden, where group 3 had higher baseline values compared to group 0 (*p* < 0.05). * indicates significant interactions where SVD burden group 3 showed steeper rates of cognitive decline compared to group 0 (*p* < 0.05). MAP: mean arterial pressure. BMI: body mass index; FSRS: Framingham Stroke Risk Score; SVD: cerebral small vessel disease.

Relative to the reference SVD burden group (SVD = 0), the highest SVD burden group had higher baseline MAP (measured approximately 22.15 years prior to the MRI Wave at mean age = 47.81; SD = 5.23; *p* = 0.03, Cohen’s f^2^ = 0.01) after correcting for age, sex, education, and MRI scanner model. However, the SVD groups did not differ in baseline BMI and FSRS, or rates of change of MAP, BMI and FSRS over this follow up period (Table 2; Figure 1).

**Table 2.** Longitudinal associations of vascular factors with SVD burden.

	MAP		BMI		Log-FSRS	
	Beta	95% CI	Beta	95% CI	Beta	95% CI
Main effect of SVD burden group						
SVD 1 vs. 0	1.75	–0.18 to 3.67	0.04	–0.63 to 0.70	0.06	–0.002 to 0.11
SVD 2 vs. 0	1.39	–0.79 to 3.57	0.06	–0.70 to 0.82	0.05	–0.02 to 0.11
SVD 3 vs. 0	**3.36**	**0.42 to 6.30^a^**	0.13	–0.89 to 1.15	0.05	–0.03 to 0.14
Interaction SVD group and time						
Time×SVD 1 vs. 0	–0.02	–0.13 to 0.08	0.01	–0.03 to 0.01	0.001	–0.002 to 0.005
Time×SVD 2 vs. 0	–0.05	–0.17 to 0.06	0.002	–0.02 to 0.02	0.001	–0.002 to 0.01
Time×SVD 3 vs. 0	–0.06	–0.21 to 0.10	–0.004	–0.03 to 0.03	0.002	–0.003 to 0.01
Interaction SVD group and time^2^						
Time^2^×SVD 1 vs. 0					–0.02	–0.04 to 0.003
Time^2^×SVD 2 vs. 0					–0.01	–0.04 to 0.01
Time^2^×SVD 3 vs. 0					0.001	–0.03 to 0.04

Note: Results of linear mixed effect models with random effects for the intercept and slope (time for MAP, BMI FSRS and time^2^ for FSRS). Vascular risk factors were assessed between Wave 3 (1991–1994) and Wave 12 (2015–2016). All models were adjusted for age at baseline, sex, education, and scanner.

MAP: mean arterial pressure; BP: blood pressure; FSRS: Framingham Stroke Risk Score; CI: confidence interval; SVD: cerebral small vessel disease.

^a^*p* < 0.05.

After adjustments for age, sex, education and MRI scanner model, relative to the reference SVD group (SVD = 0), individuals with higher SVD burden demonstrated faster linear declines in letter fluency (*p* = 0.02, Cohen’s f^2^ = 0.002) as well as linear (*p* = 0.047, Cohen’s f^2^ = 0.002) and exponential (*p* = 0.04, Cohen’s f^2^ = 0.002) declines in verbal reasoning over a time period of approximately 16.38 years (from mean age 53.58 to mean age 69.96). However, these associations did not survive Bonferroni corrections. The SVD burden groups did not differ on baseline cognitive performance or rate of cognitive decline for semantic fluency, memory, and numerical reasoning (summary statistics in Table 3; Figure 1).

**Table 3.** Longitudinal associations of cognitive performance with SVD burden.

	Letter fluency		Semantic fluency		Short-term Memory		Verbal reasoning		Numerical reasoning	
	Beta	95% CI	Beta	95% CI	Beta	95% CI	Beta	95% CI	Beta	95% CI
Main effect of SVD burden group										
SVD 1 vs. 0	0.62	–0.12 to 1.35	0.17	–0.52 to 0.87	–0.10	–0.50 to 0.30	0.52	–0.24 to 1.27	0.22	–0.68 to 1.13
SVD 2 vs. 0	0.25	–0.58 to 1.09	–0.28	–1.06 to 0.50	–0.14	–0.60 to 0.31	0.71	–0.14 to 1.57	0.12	–0.90 to 1.14
SVD 3 vs. 0	0.18	–0.95 to 1.30	0.001	–1.06 to 1.06	–0.09	–0.71 to 0.53	1.12	–0.04 to 2.28	0.24	–1.15 to 1.63
Interaction SVD group and time										
Time×SVD 1 vs. 0	–0.03	–0.07 to 0.01	0.002	–0.03 to 0.04	0.01	–0.02 to 0.03	–0.02	–0.05 to 0.02	0.01	–0.02 to 0.05
Time×SVD 2 vs. 0	–0.02	–0.07 to 0.02	0.04	–0.003 to 0.04	–0.002	–0.03 to 0.03	–0.02	–0.06 to 0.02	0.02	–0.02 to 0.06
Time×SVD 3 vs. 0	–**0.07**	–**0.13 to** –**0.01**^a^	–0.01	–0.06 to 0.05	0.01	–0.03 to 0.05	–**0.05**	–**0.11 to **–**0.001**^a^	–0.01	–0.06 to 0.05
Interaction SVD group and time^2^										
Time^2^×SVD 1 vs. 0	–0.05	–0.27 to 0.18	–0.10	–0.30 to 0.10	–0.07	–0.21 to 0.08	–0.06	–0.25 to 0.14	0.03	–0.17 to 0.23
Time^2^×SVD 2 vs. 0	–0.07	–0.32 to 0.19	–0.13	–0.36 to 0.10	–**0.21**	–**0.37 to **–**0.04**^a^	–0.16	–0.38 to 0.06	0.15	–0.08 to 0.38
Time^2^×SVD 3 vs. 0	–0.30	–0.64 to 0.05	–0.09	–0.40 to 0.22	0.08	–0.15 to 0.30	–**0.31**	–**0.60 to **–**0.01**^a^	0.24	–0.07 to 0.55

Note: Results of linear mixed effect models with random effects for the intercept and slope (time, time^2^). Cognitive tests were assessed between Wave 5 (1997–1999) and approximately 16 ± SD years later at Wave 12 (2015-2016). All models were adjusted for age at baseline, sex, education, scanner model.

CI: confidence interval; SVD: cerebral small vessel disease.

^a^*p* < 0.05.

**Figure 2.**
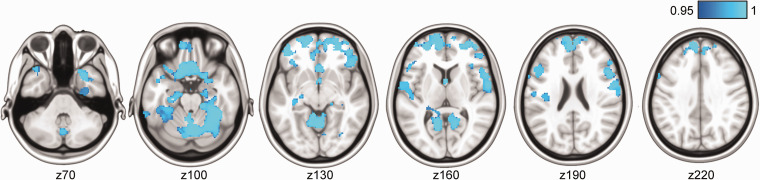
Voxel-wise associations between higher total cerebral small vessel disease (SVD) scores and lower grey matter density were primarily found cortical and hippocampal areas, shown in blue. TFCE-corrected statistical maps are overlaid on the MNI-152 template and thresholded at a 1-p value of 0.95, representing *p* < 0.05. Images are also FWE-corrected for multiple voxel-wise comparisons. Analysis was adjusted for age, sex, education, MRI scanner model, antihypertensive use, body mass index, and mean arterial pressure measured at the time of the MRI scan.

VBM analysis revealed that higher SVD burden was associated with lower GM density in several areas, including the frontal pole, superior and inferior frontal gyrus, pre- and postcentral gyrus, precuneus, frontal orbital cortex, subcallosal cortex, left Heschl’s gyrus, parahippocampal gyrus, and hippocampus (Figure 2, FWE-corrected *p* < 0.05). Together, these regions covered 9.7% of the total brain GM volume.

**Figure 3.**
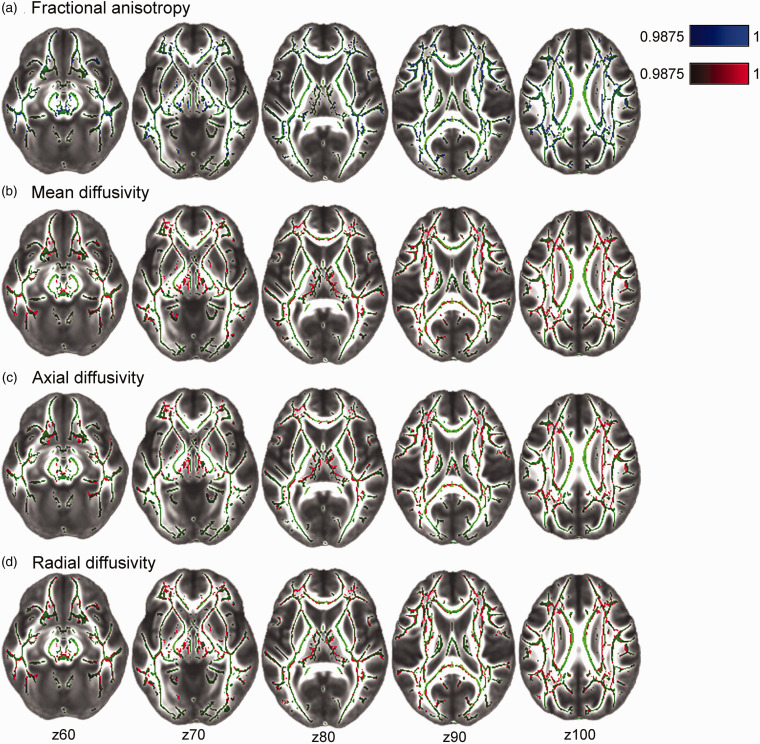
Voxel-wise associations between cerebral small vessel disease (SVD) burden and diffusion tensor imaging measures: fractional anisotropy (FA; A) mean diffusivity (MD; B); axial diffusivity (AD; C), and radial diffusivity (RD; D). Statistical maps were overlaid on the FMRIB58_FA standard image. Green tracts depict the standardized mean FA skeleton. Higher SVD burden is associated with lower FA (in blue), and higher MD, AD, RD (in red). All images are TFCE-corrected statistics thresholded at 1-p values of 0.9875, i.e. representing Bonferroni-corrected *p* < 0.0125 to adjust for multiple comparisons across 4 DTI metrics. Images are also FWE-corrected for multiple voxel-wise comparisons. Analyses were adjusted for age, sex, education, MRI scanner model, antihypertensive use, body mass index, and mean arterial pressure measured at the time of the MRI scan.

Voxel-wise TBSS analyses revealed that higher SVD MRI burden was associated with lower FA, and higher MD, RD, and AD (Bonferroni-corrected *p* < 0.0125) throughout several projection and commissural WM tracts such as the corpus callosum, cingulum, corona radiata, and longitudinal fasciculus (Figure 3). Associations were distributed across 17.0%, 16.7%, 15.4% and 23.8% of total WM tract skeletons for FA, MD, RD and AD, respectively. These results remained after a voxel-wise correction for WMH masks.

**Figure 4.**
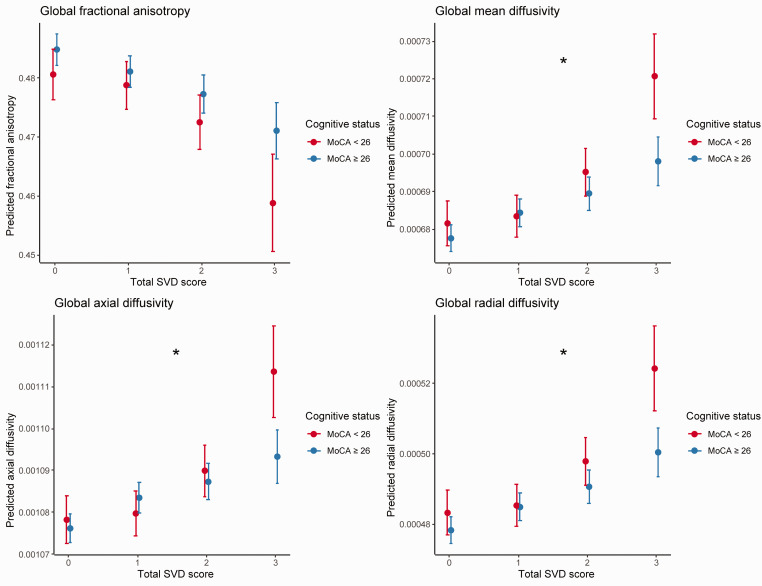
Interaction between cognitive status and cerebral small vessel disease burden on global diffusion metrics. Plots depict significant interactions between cognitive status (Montreal Cognitive Assessment [MoCA]<26 vs. ≥26) and total SVD scores (0-3) on mean diffusivity, axial diffusivity, and radial diffusivity (all *p<*.05; depicted with *), but not for fractional anisotropy. The association between SVD burden and DTI parameters is more pronounced in individuals with cognitive impairments, reflected by MoCA < 26, and especially in the most severe SVD burden group. MoCA: Montreal Cognitive Assessment; SVD: cerebral small vessel disease.

We observed an interaction between SVD burden (0-3) and cognitive status at the MRI Wave (MoCA < 26 vs. MoCA ≥ 26; (*F*_3,608_ = 2.14, *p* = 0.007, Cohen’s f^2^ = 0.13). Post-hoc univariate comparisons revealed that this interaction effect was driven by MD (*F*_3,608_ = 3.27, *p* = 0.02, Cohen’s f^2^ = 0.13), AD (*F*_3,608_ = 3.72, *p* = 0.01, Cohen’s f^2^ = 0.14), and RD (*F*_3,608_ = 2.72, *p* = 0.04, Cohen’s f^2^ = 0.12), but not FA (*F*_3,608_ = 1.03, *p* = 0.38) or GM (*F*_3,608_ = 0.97, *p* = 0.41). For each of these interactions, the association of SVD burden with poor WM microstructure was more pronounced in individuals with cognitive impairments (reflected by MoCA < 26, Figure 4).

## Discussion

This study characterized late-life SVD burden across a large sample of community-dwelling older adults and has demonstrated several associations with up to 25-year retrospective trajectories of vascular risk factors, cognitive performance, and brain microstructure. We showed that individuals with severe SVD burden at the MRI Wave (mean age 70) presented with elevated mean arterial pressure about 20 years prior to their MRI (Wave 3, mean age 48 years). We also found that individuals with higher SVD burden in older age had relatively faster rates of decline in letter fluency and verbal reasoning performance from mid-to-late life (i.e. from mean ages 54 to 70 years). In addition, at the time of MRI, we revealed concurrent associations of SVD burden with widespread reductions in GM density as well as hallmark indicators of WM structural integrity, reflected by reduced FA and increased diffusivity. Importantly, the negative associations between SVD and diffusivity were stronger in individuals with cognitive impairments (MoCA < 26). Overall, our findings could have important implications for optimizing strategies for maintaining brain and cognitive health during the lifespan.

While previous studies have revealed associations between higher midlife blood pressure and late-life SVD in normal aging, these have only examined either WMH, CMB, or brain infarcts individually.^7,9^ Here, we show that higher MAP in midlife (mean age 48, Wave 3) is associated with severe total SVD burden approximately 20 years later (mean age 70, MRI Wave), measured as a combination of four key MRI markers of SVD pathology.^4^ Our results thus indicate that elevated midlife MAP can have more diffuse effects on cerebrovascular health that go beyond the single associations with separate SVD features. Our findings are in line with previous studies showing that hypertension exacerbates SVD-related pathologies in aging individuals.^1,25^ We found no evidence for the contribution of other midlife vascular risk factors (BMI or stroke risk scores) to late-life SVD burden, suggesting that midlife blood pressure may particularly aggravate SVD-related injuries and therefore may serve as the most important modifiable risk factor for SVD.^26^ This study therefore adds to the accumulating evidence emphasizing the need for early prevention strategies with a focus on blood pressure management.^5–7,9^ However, individuals with SVD are not always hypertensive, and given that our results are observational, we cannot determine causality, or rule out reverse causation.^27^ Therefore, the potential clinical consequences of our findings warrant further investigation.

WMH, infarcts, CMBs have each individually been linked to cognitive decline and dementia, although the association with EPVS is less clear.^28^ Two previous studies demonstrated steeper rates of decline of global cognition and executive function, however these links were only established in hypertensive patients^29^ and symptomatic SVD.^30^ Our findings show for the first time that, in adults without a diagnosis of stroke or dementia, higher SVD burden in older ages relates to faster rates of decline in letter fluency and verbal reasoning performance during the preceding 16 years. As SVD features are common in elderly individuals and increase risk of Alzheimer’s and vascular dementia,^28^ these results highlight the importance of investigating how SVD links to preclinical cognition impairments. We also made two key observations: first, that these associations with cognitive decline were only present in participants with ≥3 SVD features, which occurred in 10% of the total sample. Similar to an earlier study,^31^ there appeared to be a threshold effect, where cognitive dysfunctions became noticeable only in those with moderate-to-severe SVD burden. Second, the effects of SVD on cognition were generally small, and did not survive strict corrections for multiple-comparisons. While it is possible that larger studies could confirm our observed associations at an uncorrected *p* < 0.05, accumulating evidence suggests that there is a large inter-individual variability in the effects of SVD burden on cognition, both with regards to the affected domains and severity.^25,32^ Overall we observed that despite their high SVD burden, individuals displayed relatively modest increases in rates of cognitive decline. This could partially be explained by the concept of reserve, which refers to the ability to alleviate the effects of neuropathology through variations in brain structure (i.e., brain reserve) or adaptability of functional processes (i.e. cognitive reserve).^33^ Brain reserve is generally considered to be a less dynamic construct, whereas cognitive reserve is thought to be more adjustable through life experiences (e.g., by increasing cognitive engagement, physical activity, leisure activities)^33^ and therefore may serve as viable target to improve clinical management of the consequences of SVD.^8,25^

Additionally, we found specific GM and WM correlates of current SVD-related burden. More specifically, we identified GM density reductions predominantly in the medial-frontal, orbito-frontal, and medial-temporal regions. This pattern of cortical atrophy has been recognized in earlier studies examining WMH or lacunes alone.^34,35^ These associations were most pronounced in the medial temporal and hippocampal regions, which are traditionally linked to AD pathology.^36^ This is especially important given the growing recognition of the prevalence of SVD and cerebrovascular dysfunction in AD.^37^

Grey matter atrophy may result from secondary neurodegenerative processes elicited by SVD-related damage to the WM tracts that disrupt the connections between remote brain regions.^1,25^ Indeed, in this study we also noted widespread alterations of WM tracts in relation to SVD burden, reflected by decreased FA, and increased MD, AD, and RD, all of which are established markers of WM microstructural damage in ageing and dementia.^20^ It is noteworthy that these associations remained after adding WMH masks as voxel-wise confounds, indicating that WMH alone did not explain these associations, but that SVD severity beyond WMH instead concurs with white matter microstructural abnormalities.^38^ These findings thus underscore that total SVD burden scores more comprehensively characterize the consequences of SVD than the sole consideration of single MRI features.^3,4^ We also revealed that, relative to cognitively healthy adults, those with cognitive impairments (MoCA < 26) had more pronounced negative associations between SVD and WM diffusivity, highlighting the central role of WM microstructure in the emergence of cognitive impairments.^32,38^ Altogether, we demonstrate that the accumulation of SVD-related damage may manifest clinically through diffuse effects on WM tracts throughout the brain, and relate to gross changes in brain regions distant from the initial lesion site.^1,25^

The importance of managing midlife vascular health to maintain cognition in late life has been demonstrated previously in the Whitehall II and other observational cohorts.^5–7,9,39^ Our findings suggest that SVD may also play an important part in this relationship. However, the mechanisms linking SVD-associated injuries with our observed changes in mid-life blood pressure, diffuse brain microstructural alterations, and subsequent cognitive dysfunction remain to be elucidated.^27^ One potential mediator in this pathway which could provide a direct link between peripheral and central vascular damage may be large artery stiffening, which has been associated with SVD burden,^40^ midlife hypertension, damage to the blood-brain barrier, subsequent brain atrophy, and cognitive impairment.^39,41^ Future longitudinal studies which include multiple assessments of SVD features would help unravel these complex mechanisms and temporal dynamics in further detail.

Strengths of the current study include the detailed examination of participants for up to 25 years, including repeated assessments of vascular risk and cognitive functions and the acquisition of advanced and multi-modal MRI scans in old age, allowing for a comprehensive characterization of total SVD burden. However, several limitations should be noted. First, the generalizability of our findings is limited as participants of the Whitehall II study are predominantly Caucasian, generally more highly educated and healthier as compared to nationally representative samples, and only 20.7% individuals were female in this study.^42,43^ Second, in contrast to most of the previous work that investigated global SVD burden among patient groups (e.g., stroke or hypertensive populations),^3,11,29,30^ our sample was relatively healthy, partially due to our selection criteria (e.g., MRI compatibility; no gross brain abnormalities).Third, T2-weighted images were not acquired in this cohort, so instead we incorporated both T1-weighted and FLAIR images to distinguish EPVS and lacunes. This approach is in accordance with the STRIVE criteria and has been adopted by previous studies.^44,45^ However, as T2-weighted images allow for an easier detection of cavitation relatively to FLAIR images, we note that the possibility that approach may have led to misclassification errors.^2,46,47^ Fourth, the prevalence of CMB was relatively high in this study (32%) as compared to participants of similar age ranges in other healthy cohort studies (10–30%).^48–50^ Prevalence of microbleeds increases considerably with magnetic field strength.^51^ The fact that most of the previous studies were performed on 1.5 T scanners^48,50^ and our study used a 3 T scanner may partially explain this discrepancy. Nonetheless, although we incorporated previously established criteria as well as a consensus strategy to rate CMBs and to recognize potential mimics,^52,53^ we cannot exclude the possibility of having included some false positives. Lastly, as in other studies,^3,4,10,11^ the distribution of SVD burden in this sample was skewed, with low frequency of severe SVD. Together, this may have resulted in an underestimation of the strength of our observed associations and may in part explain why our observed effects were only small-to-medium. We therefore emphasize the need to replicate our findings in diverse cohort studies.

We demonstrated longitudinal associations of late-life SVD burden with vascular and cognitive functioning. Importantly, we observed that midlife blood pressure (mean age of 48) may contribute to SVD burden 20 years later (mean age 70). Furthermore, SVD burden related to slightly faster rates of cognitive decline, together with more pronounced and widespread differences in GM and WM microstructure. Together, our findings further emphasize the importance of midlife vascular health to maintain brain structure and function.

## Supplemental Material

sj-pdf-1-jcb-10.1177_0271678X211048411 - Supplemental material for Association of cerebral small vessel disease burden with brain structure and cognitive and vascular risk trajectories in mid-to-late life Click here for additional data file. Supplemental material, sj-pdf-1-jcb-10.1177_0271678X211048411 for Association of cerebral small vessel disease burden with brain structure and cognitive and vascular risk trajectories in mid-to-late life by Michelle G Jansen, Ludovica Griffanti, Clare E Mackay, Melis Anatürk, Luca Melazzini, Ann-Marie G de Lange, Nicola Filippini, Enikő Zsoldos, Kim Wiegertjes, Frank-Erik de Leeuw, Archana Singh-Manoux, Mika Kivimäki, Klaus P Ebmeier and Sana Suri in Journal of Cerebral Blood Flow & Metabolism
