# Supplementary material for: Association of cerebral small vessel disease burden with brain structure and cognitive and vascular risk trajectories in mid-to-late life
Source: J Cereb Blood Flow Metab. 2021 Oct 5;42(4):600–12. doi: 10.1177/0271678X211048411 (PMC8943617; doi:10.1177/0271678X211048411)
Supplement: sj-pdf-1-jcb-10.1177_0271678X211048411 - Supplemental material for Association of cerebral small vessel disease burden with brain structure and cognitive and vascular risk trajectories in mid-to-late life [file sj-pdf-1-jcb-10.1177_0271678X211048411.pdf]

Supplementary material

Association of cerebral small vessel disease burden with brain structure and cognitive and vascular risk trajectories in mid-to-late life

Michelle G Jansen[1,2], Ludovica Griffanti[3,4], Clare E Mackay[3,4], Melis Anatürk[3,5], Luca Melazzini[4,6], Ann-Marie G de Lange[3,7], Nicola Filippini[8], Enikő Zsoldos[3,4], Kim Wiegertjes[2], Frank-Erik de Leeuw[2], Archana Singh-Manoux[9,10], Mika Kivimäki[9], Klaus P Ebmeier[3], Sana Suri[3,4]

[1]Donders Centre for Cognition, Donders Institute for Brain, Cognition and Behaviour, Radboud University, Nijmegen, the Netherlands

[2]Department of Neurology, Donders Institute for Brain, Cognition and Behavior, Radboud University Medical Center, Nijmegen, the Netherlands

[3]Department of Psychiatry, University of Oxford, United Kingdom

[4]Wellcome Centre for Integrative Neuroimaging (Oxford Centres for Functional MRI of the Brain & Human Brain Activity) Nuffield Department of Clinical Neurosciences & Psychiatry, University of Oxford, United Kingdom

[5]Centre for Medical Image Computing, Department of Computer Science, University College London, United Kingdom

[6]Department of Biomedical Sciences for Health, Università degli Studi di Milano, Milan, Italy

[7]Department of Psychology, University of Oslo, Norway

[8]IRCCS San Camillo Hospital, Venice, Italy

[9]Department of Epidemiology and Public Health, University College London, United Kingdom

[10]Université de Paris, INSERM, U1153, Epidemiology of Ageing and Neurogenerative diseases, Paris, France

Corresponding author

Michelle G Jansen, Donders Centre for Cognition, Donders Institute for Brain, Cognition and Behaviour, Radboud University, P.O. Box 9104, 6500 HE, Nijmegen, the Netherlands.

Email: m.jansen@donders.ru.nl.

Overview of supplementary materials

Supplementary methods

Description of cohort profile

Description of SVD ratings on MRI

MRI pre-processing steps

Study variables

Description of linear mixed effect models

Supplementary tables

Table S1. MRI acquisition parameters

Table S2. Participant characteristics of the Whitehall II study

Table S3. Included vs. complete sample of Whitehall II Imaging Sub-study

Supplementary figures

Figure S1. Flowchart of subject inclusion

Figure S2. Venn diagram of cerebral small vessel disease MRI score

Supplementary methods

Description of cohort profile

The Whitehall II study targeted all civil servants that worked in the London offices of 20 Whitehall departments between 1985–1988, established by University College London. The initial Wave included 10 308 British Civil servants (6895 men), aged 35–55 years. Whitehall II Study participants have received detailed clinical follow-ups for up to 30 years at 5-year intervals (1991-1994, Wave 3; 1997-1999, Wave 5; 2002-2004, Wave 7; 2007-2009, Wave 9; 2012-2013, Wave 11); 2015-2016, Wave 12). Since the inception of the Whitehall II study, the retention rate for this cohort has been relatively high; about 87% of Wave 9 participants returned for the follow-up at Wave 11. The Whitehall II Imaging-Sub study randomly selected 774 participants aged 60-85 years from the Whitehall II Wave 11 cohort for multi-modal brain MRI work-up and cognitive tests at the University of Oxford. For the Imaging Sub-study, participants were included with contraindications to MRI scanning (e.g., particular metallic implants) or who were unable to travel to Oxford without assistance.[1, 2]

Description of SVD ratings on MRI

Periventricular and deep white matter hyperintensities (WMH) were rated by trained raters (C.L.A., A.G.T., V.V.; see acknowledgments) on FLAIR images using the Fazekas scale, providing a score between 0-3 depending on the severity of WMH in the corresponding brain areas.[3] Enlarged perivascular spaces (EPVS) and lacunes were rated by an experienced rater (M.G.J.) following extensive training, and in consensus with other experienced raters (S.S., L.M.). Lacunes were rated using both T1-weighted and FLAIR images, following established criteria to distinguish lacunes from EPVS.[4, 5] The intra-rater reliability for lacune ratings indicated high similarity, as reflected by an intraclass correlation (ICC) of 0.91, based on a random sample of 25 participants. EPVS were assessed in the basal ganglia on T1-weighted images using the validated qualitative EPVS rating scale, as T2-weighted images were not acquired in this cohort.[6] The ICC for EPVS was 0.85, based on a random sample of 30 participants, indicating good intra-rater reliability. We used a semi-automatic detection method to identify possible cerebral microbleeds (CMBs), based on the radial symmetry transform.[7] Subsequently, one experienced rater (S.S.) evaluated all possible CMBs using previously established criteria[7, 8], and in consensus with a clinical psychiatrist (K.P.E.). The intra-rater reliability yielded excellent results (ICC = 0.92, based on a random sample of 100 participants).

MRI pre-processing steps

MRI scans were analysed using FMRIB Software Library v6.0 (FSL; https://fsl.fmrib.ox.ac.uk/).[9]

T1 images were bias corrected, brain extracted using FSL-ANAT and segmented using FSL-FAST to provide estimates of grey matter (GM, white matter (WM) and total brain volume (TBV).[10] All segmentations were visually inspected to ensure quality.

Diffusion-weighted images were pre-processed using FMRIB's diffusion toolbox.[11] Briefly, after applying motion and eddy current corrections with FSL-TOPUP, diffusivity maps for each metric was extracted using DTIFit and aligned into standard space using FMRIB's Nonlinear Registration Tool (FNIRT).

FLAIR scans were used to extract WMH using the Brain Intensity AbNormality Classification Algorithm (BIANCA).[12] This algorithm uses both intensity features (provided from FLAIR, T1 images, and fractional anisotropy) and spatial features to classify all voxels. BIANCA was initially trained on manually segmented WMH masks of participants who were scanned on the Prisma scanner (N = 24), Verio scanner (N=24), and an independent sample from the UK Biobank Study (N = 12) to avoid scanner-dependent bias effects. This approach was specifically designed to improve the consistency of BIANCA output, and has been shown to result in better intra- and inter-rater reliability relatively to manual segmentations.[12-14]

Additional information on pre-processing was mentioned previously.[1, 15] With regards to **harmonization between scanners**, several of the aforementioned pre-processing steps were specifically incorporated to minimize scanner effects (i.e. bias correction on T1 images, and training BIANCA on the Prisma, Verio, and an independent sample of the UK Biobank). In addition, we have compared volumetric measures between the Prisma and Verio scanner in previous work, where measures of GM and CSF were relatively increased at the Prisma scanner, and measures of WM were relatively increased at the Verio scanner.[16] To ensure that our results were not affected by scanner effects, we also included scanner as confounding variable in all of our analyses.

Study variables

The **FSRS** was based on age, sex, systolic blood pressure, use of antihypertensive medications, diabetes mellitus, current smoking, current or history of atrial fibrillation, left ventricular hypertrophy, and current or history of cardiovascular disease.[17] These measurements were obtained using both questionnaires and clinical assessments, using standard operating protocols, as described previously.[18, 19] **Blood pressure** measurements were obtained in sitting position after five minutes rest; the average of two measurements was used for further analysis. The use of **antihypertensive medication** was self-reported (e.g., diuretics, beta blockers, angiotensin-converting enzyme inhibitors, and calcium channel blockers). **Diabetes** was defined by having a fasting glucose level of ≥ 7.0 mml/L or a 2hr post-load glucose level of ≥ 11.1 mml/L, based on glucose measurements obtained from venous blood; self-reported diabetes diagnosed by a doctor or use of diabetes medication.[20] **Smoking behaviour** was self-reported (current, past/no smoking). A standard electrocardiogram analysis combined with manual review and Minnesota code classification system for electrocardiographic findings was used to identify **atrial fibrillation** and **left ventricular hypertrophy**.[21] **Cardiovascular disease** was evaluated using corroborated records from the general practitioner, hospital, and electrocardiogram and angiogram examinations at Wave 1, 3 and 5. Subsequently, the FSRS was computed using the beta coefficients of the Cox proportional hazards regression model in the Framingham Study, to indicate an individual's 10-year risk of stroke.[22]

The longitudinal test battery of the Whitehall II cohort includes several cognitive tests, proven to be sensitive to detect changes in cognitive functions in this study population.[23] The complete test battery took 30 minutes to complete. To measure **letter fluency**, participants were instructed to recall as many words beginning with an "S" within one minute. For **semantic fluency**, participants were given similar instructions, but instead needed to recall as many animal names. **Short-term memory** was evaluated by initially presenting a list of 20 one or two syllable words at two seconds intervals. Subsequently, participants were asked to recall as many of the word list, within two minutes. The Alice Heim 4-I test composes 65 verbal and mathematical reasoning items with increasing difficulty (e.g., where participants had to identify certain patterns or rules), covering **verbal and numerical reasoning**.[24] Participants were given 10 minutes to complete the test. Besides this, the test battery also included the Mill Hill vocabulary test[25] and the Mini Mental State Examination[26], however these tests were not included in the present study due to the observed ceiling effects.

Additional information on the vascular and cognitive study variables was mentioned previously.[16, 23, 27, 28]

Description of linear mixed effect models

To investigate the association between the cerebral small vessel disease (SVD) MRI score and trajectories of vascular risk and cognitive performance over 25 years, we employed the following equation for each dependent variable of interest (V):

$$V_{ij} = \beta_0 + \beta_1 time_{ij} + \beta_2 time_{ij}^2 + \beta_3 SVD_{ij} + \beta_4 SVD_{ij} time_{ij} + \beta_5 SVD_{ij} time_{ij}^2 + \beta_6 X_{1i} + \beta_7 X_{2i} + \beta_8 X_{2i} time_{ij} + \beta_9 X_{1i} time_{ij}^2 + U_{0i} + U_{1i} time_{ij} + e_{ij}$$

V_{ij} is the dependent variable of interest of the ith participant at the jth occasion,
$time_{ij}$ is years since the baseline measurement between 1995-1999 for the ith participant at the jth occasion,
$time^2_{ij}$ is the orthogonalized polynomial quadratic time term for the ith participant at the jth occasion,
SVD_i is the total SVD score obtained during the Whitehall Imaging Sub-study of the ith participant,
X_{1i} is the covariate for scanner model (Prisma vs. Verio) for the ith participant,
X_{2i} is a vector of covariates (age at baseline, sex, education) for the ith participant,
U_{0i} is the random intercept, U_{1i} is the random slope, and e_{ij} is the residual.

The dependent variables of interest focused on vascular risk and cognitive performance.

Vascular risk was defined using the Framingham Stroke Risk Score (FSRS) and mean arterial pressure (MAP). Due to the skewed distribution of the residuals, FSRS was log-transformed.

Cognitive performance was covered for the following domains: letter fluency, semantic fluency, verbal reasoning, numerical reasoning, and global cognition.

Vascular risk factors were measured at six waves (j= 1, 2, 3, 4, 5, 6), whereas measures for cognitive performance were included from five waves (j = 1, 2, 3, 4, 5).

Parameter estimates were obtained with the Maximum Likelihood method from the *nlme* in R version 3.6.1.

Main effects of SVD burden are indicative of whether SVD burden scores (1-3) differed from the reference score (no burden; 0) on the variable of interest at baseline. The interaction of SVD burden with time indicates whether a higher SVD burden (1-3) is associated with different longitudinal trajectories (i.e., slopes) of the respective variable of interest as compared to the reference score. To allow for individual rates of change of the dependent variables over time for each participant, we fitted the intercept and slope as random effects. We implemented a continuous autoregressive moving-average correlation structure to consider repeated measures for each individual.

Supplementary tables

Table S1. MRI acquisition parameters

		TR (ms)	TE (ms)	TI (ms)	Flip angle (°)	Field of view (mm)	Matrix (voxels)
**T1-weighted**							
	Verio	2530	1.79/3.65/ 5.51/7.37	1380	7	256	1.0x1.0x1.0
	Prisma	1900	3.97	904	8	192	1.0x1.0x1.0
**FLAIR**							
	Verio	9000	73	2500	150	220	0.9x0.9x3.0
	Prisma	9000	73	2500	150	220	0.4x0.4x3.0
**T2*-weighted**							
	Verio	36	30	-	15	220	0.7x0.7x1.5
	Prisma	1230	13.4	-	25	206	0.8x0.8x5.0
**DWI**							
	Verio	8900	91.2	-	-	192	2.0x2.0x2.0
	Prisma	8900	91	-	-	192	2.0x2.0x2.0
**B0**							
	Verio	8900	91.2	-	-	192	2.0x2.0x2.0
	Prisma	8900	91	-	-	192	2.0x2.0x2.0

Abbreviations: FLAIR: fluid-attenuated inversion recovery; DWI: diffusion-weighted imaging; TR: repetition time; TE: echo time; TI: inversion time.

Table S2. Sample characteristics for the 623 participants of the Whitehall II study

	Wave 3 (1991-1994)		Wave 5 (1997-1999)		Wave 7 (2002-2004)		Wave 9 (2007-2009)		Wave 11 (2012-2013)		Wave 12 (2015-2016)	
**General characteristics**	N	Mean (SD or median (IQR)	N	Mean (SD or median (IQR)	N	Mean (SD or median (IQR)	N	Mean (SD or median (IQR)	N	Mean (SD or median (IQR)	N	Mean (SD or median (IQR)
Age	623	47.81 (5.23)	608	53.58 (5.22)	609	59.07 (5.20)	620	64.01 (5.21)	623	68.10 (5.22)	610	71.30 (5.23)
**Vascular risk factors**												
MAP	608	91.63 (9.71)	585	90.64 (11.24)	603	90.08 (10.90)	619	87.94 (10.66)	623	88.64 (10.46)	606	89.19 (10.78)
BMI	607	24.80 (3.32)	517	25.26 (3.70)	603	26.21 (3.91)	620	26.29 (4.10)	623	26.28 (4.11)	606	26.39 (4.09)
FSRS	588	3 (3-4)	869	3 (3-4)	594	4 (3-6)	603	5 (4-7)	602	6 (5-10)	578	7 (5-11)
**Cognitive performance**												
Letter fluency	290	17.84 (4.17)	560	17.72 (4.19)	593	16.87 (3.81)	615	16.13 (3.64)	622	16.09 (4.01)	602	15.58 (4.28)
Semantic fluency	290	16.69 (3.44)	559	17.21 (4.00)	596	16.56 (3.59)	617	15.93 (3.58)	622	15.77 (3.59)	602	15.80 (3.54)
Verbal reasoning	289	25.17 (4.49)	561	25.30 (4.23)	596	24.10 (4.35)	618	23.79 (4.58)	622	23.72 (4.59)	603	23.98 (4.77)
Numerical reasoning	289	24.44 (5.28)	561	24.39 (5.16)	596	23.31 (5.11)	618	22.89 (5.42)	622	22.75 (5.53)	603	21.84 (5.64)
Memory	288	6.15 (2.15)	558	7.31 (2.30)	595	7.35 (2.17)	618	6.59 (2.19)	622	6.58 (2.24)	599	5.68 (2.20)

Abbreviations: SD: standard deviation; IQR: interquartile range; MAP: mean arterial pressure; FSRS: Framingham Stroke Risk Score.

Table S3. Included vs. complete sample of Whitehall II Imaging Sub-study at MRI wave

	Included sample	Complete sample	P-value
Number of participants	623	775	
Age, mean (SD)	69.96 (5.18)	69.81 (5.19)	0.59
Female, N (%)	129 (21%)	150 (19%)	0.58
Education, mean (SD)	14.11 (3.05)	14.06 (3.06)	0.74
MoCA, median (IQR)	28 (26-29)	28 (26-29)	0.74
MAP, mean (SD)	98.84 (11.82)	98.79 (11.70)	0.95
BMI, mean (SD)	25.96 (4.14)	26.15 (4.17)	0.38

Abbreviations: MoCA: Montreal cognitive assessment. MAP: mean arterial pressure. BMI: body mass index. Differences in characteristics were compared using independent *t*-test, Chi-square test or Mann-Whitney U test where appropriate. Data on MAP was missing in 4 (0.05%) from the complete sample.

Supplementary figures

Figure S1. Flowchart of subject inclusion

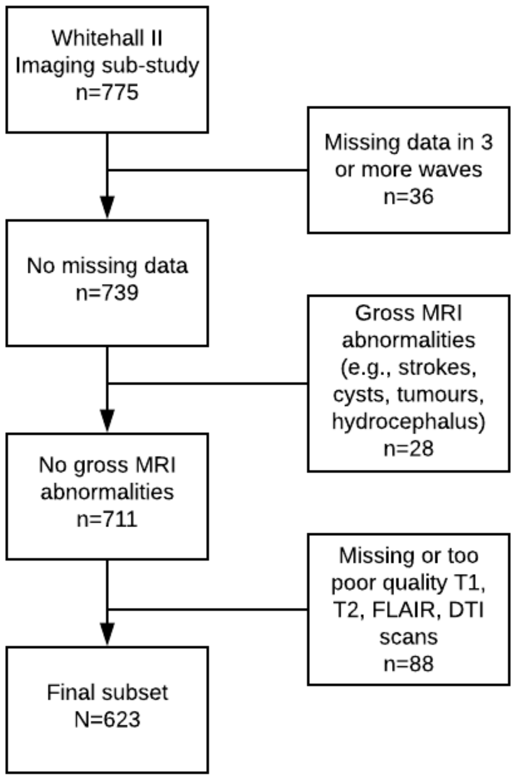

Figure S2. Venn diagram of cerebral small vessel disease MRI score

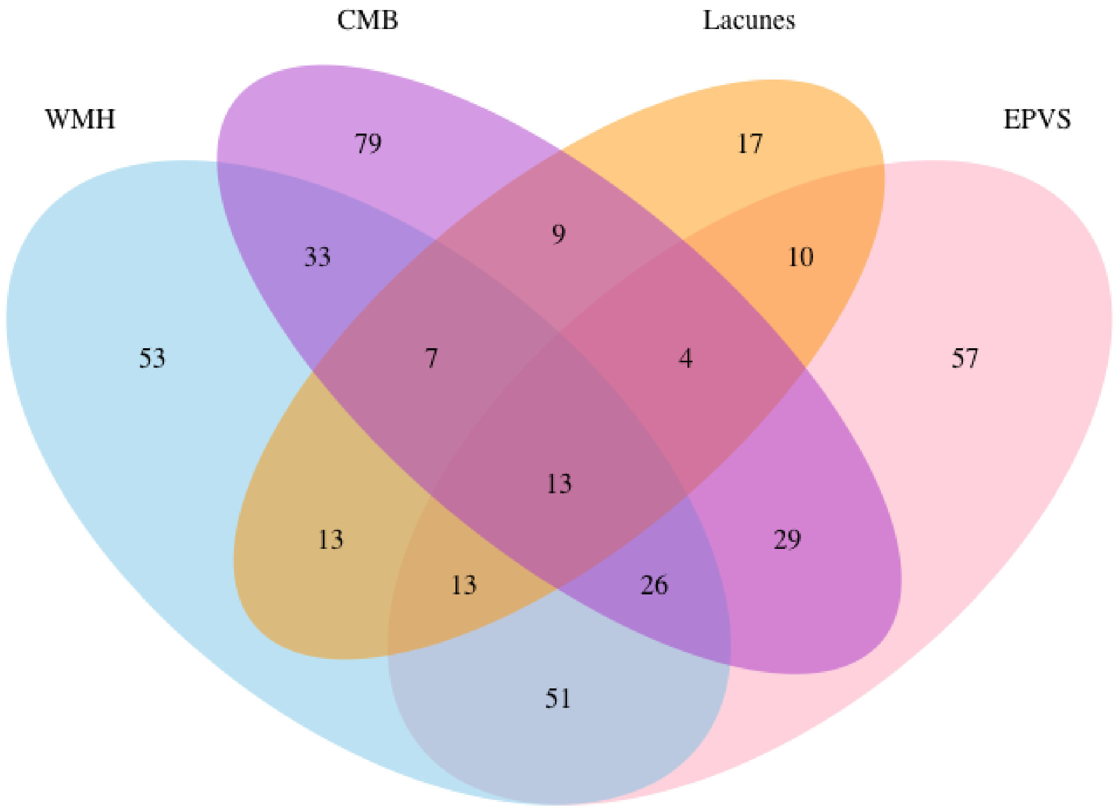

Abbreviations: WMH: white matter hyperintensities; CMB: cerebral microbleeds; EPVS: enlarged perivascular spaces. Numbers depict how many participants were within a certain category. Only 13 participants demonstrated all four features of cerebral small vessel disease on MRI.

Supplemental references

1. Filippini N, Zsoldos E, Haapakoski R, et al. Study protocol: the Whitehall II imaging sub-study. *BMC Psychiatry* 2014; 14: 159.

2. Marmot M and Brunner E. Cohort profile: the Whitehall II study. *Int J Epidemiol* 2005; 34: 251-256.

3. Fazekas F, Chawluk JB, Alavi A, et al. MR signal abnormalities at 1.5 T in Alzheimer's dementia and normal aging. *Am J Roentgenol* 1987; 149: 351-356.

4. Duering M, Csanadi E, Gesierich B, et al. Incident lacunes preferentially localize to the edge of white matter hyperintensities: insights into the pathophysiology of cerebral small vessel disease. *Brain* 2013; 136: 2717-2726.

5. Benjamin P, Trippier S, Lawrence AJ, et al. Lacunar infarcts, but not perivascular spaces, are predictors of cognitive decline in cerebral small-vessel disease. *Stroke* 2018; 49: 586-593.

6. Potter GM, Chappell FM, Morris Z, et al. Cerebral perivascular spaces visible on magnetic resonance imaging: development of a qualitative rating scale and its observer reliability. *Cerebrovascular diseases* 2015; 39: 224-231.

7. Kuijf HJ, Brundel M, de Bresser J, et al. Semi-automated detection of cerebral microbleeds on 3.0 T MR images. *PLoS One* 2013; 8: e66610.

8. Linn J. Imaging of Cerebral Microbleeds. *Clin Neuroradiol* 2015; 25: 167-175.

9. Jenkinson M, Beckmann CF, Behrens TE, et al. Fsl. *Neuroimage* 2012; 62: 782-790.

10. Zhang Y, Brady M and Smith S. Segmentation of brain MR images through a hidden Markov random field model and the expectation-maximization algorithm. *IEEE Trans Med Imaging* 2001; 20: 45-57.

11. Smith SM, Jenkinson M, Johansen-Berg H, et al. Tract-based spatial statistics: voxelwise analysis of multi-subject diffusion data. *Neuroimage* 2006; 31: 1487-1505.

12. Griffanti L, Jenkinson M, Suri S, et al. Classification and characterization of periventricular and deep white matter hyperintensities on MRI: A study in older adults. *NeuroImage* 2018; 170: 174-181.

13. Bordin V, Bertani I, Mattioli I, et al. Integrating large-scale neuroimaging research datasets: Harmonisation of white matter hyperintensity measurements across Whitehall and UK Biobank datasets. *Neuroimage* 2021; 237: 118189.

14. Griffanti L, Zamboni G, Khan A, et al. BIANCA (Brain Intensity AbNormality Classification Algorithm): A new tool for automated segmentation of white matter hyperintensities. *Neuroimage* 2016; 141: 191-205.

15. Suri S, Chiesa ST, Zsoldos E, et al. Associations between arterial stiffening and brain structure, perfusion, and cognition in the Whitehall II Imaging Sub-study: A retrospective cohort study. *PLoS Med* 2020; 17: e1003467.

16. Zsoldos E, Mahmood A, Filippini N, et al. Association of midlife stroke risk with structural brain integrity and memory performance at older ages: a longitudinal cohort study. *Brain Comm* 2020; 2

17. Wolf PA, D'Agostino RB, Belanger AJ, et al. Probability of stroke: a risk profile from the Framingham Study. *Stroke* 1991; 22: 312-318.

18. Kaffashian S, Dugravot A, Brunner EJ, et al. Midlife stroke risk and cognitive decline: a 10-year follow-up of the Whitehall II cohort study. *Alzheimer's & Dementia* 2013; 9: 572-579.

19. Kivimäki M, Shipley MJ, Allan CL, et al. Vascular risk status as a predictor of later-life depressive symptoms: a cohort study. *Biol Psychiatry* 2012; 72: 324-330.

20. Association AD. Report of the expert committee on the diagnosis and classification of diabetes mellitus. *Diabetes Care* 2003; 26: s5-s20.

21. Blackburn H, Prineas RJ and Crow RS. *The Minnesota Code: manual for electrocardiographic findings: standards and procedures for measurement and classification*. Bristol, UK: John Wright, 1982.

22. D'Agostino RB, Wolf PA, Belanger AJ, et al. Stroke risk profile: adjustment for antihypertensive medication. The Framingham Study. *Stroke* 1994; 25: 40-43.

23. Singh-Manoux A, Kivimaki M, Glymour MM, et al. Timing of onset of cognitive decline: results from Whitehall II prospective cohort study. *BMJ* 2012; 344: d7622.

24. Heim A. The AH4 group test of intelligence. *Windsor: NFER-Nelson* 1970

25. Raven JC. *Guide to Using the Mill Hill Vocabulary Scale with Progressive Matrices (1938)*. HK Lewis, 1954.

26. Folstein MF, Folstein SE and McHugh PR. "Mini-mental state": a practical method for grading the cognitive state of patients for the clinician. *J Psychiatr Res* 1975; 12: 189-198.

27. Singh-Manoux A, Britton AR and Marmot M. Vascular disease and cognitive function: evidence from the Whitehall II Study. *J Am Geriatr Soc* 2003; 51: 1445-1450.

28. Suri S, Topiwala A, Chappell MA, et al. Association of midlife cardiovascular risk profiles with cerebral perfusion at older ages. *JAMA Netw* 2019; 2: e195776-e195776.
